# Defined microbial communities and their soluble products protect mice from *Clostridioides difficile* infection

**DOI:** 10.1038/s42003-024-05778-6

**Published:** 2024-01-27

**Authors:** Katya Douchant, Shu-Mei He, Curtis Noordhof, Jill Greenlaw, Sarah Vancuren, Kathleen Schroeter, Emma Allen-Vercoe, Calvin Sjaarda, Stephen J. Vanner, Elaine O. Petrof, Prameet M. Sheth, Mabel Guzman

**Affiliations:** 1 The Gastrointestinal Disease Research Unit (GIDRU), Kingston Health Sciences Center, Kingston, K7L2V7 ON Canada; 2 Department of Biomedical and Molecular Sciences, Queen’s University, Kingston, K7L3N6 ON Canada; 3 Department of Molecular and Cellular Biology, University of Guelph, Guelph, N1G2W1 ON Canada; 4 Division of Microbiology, Kingston Health Sciences Center, Kingston, K7L2V7 ON Canada; 5 Department of Pathology and Molecular Medicine, Queen’s University, Kingston, K7L3N6 ON Canada

**Keywords:** Clostridium difficile, Bacterial infection

## Abstract

*Clostridioides difficile* is the leading cause of antibiotic-associated infectious diarrhea. The development of *C.difficile* infection is tied to perturbations of the bacterial community in the gastrointestinal tract, called the gastrointestinal microbiota. Repairing the gastrointestinal microbiota by introducing lab-designed bacterial communities, or defined microbial communities, has recently shown promise as therapeutics against *C.difficile* infection, however, the mechanisms of action of defined microbial communities remain unclear. Using an antibiotic- *C.difficile* mouse model, we report the ability of an 18-member community and a refined 4-member community to protect mice from two ribotypes of *C.difficile* (CD027, CD078; *p* < 0.05). Furthermore, bacteria-free supernatant delivered orally to mice from the 4-member community proteolyzed *C.difficile* toxins in vitro and protected mice from *C.difficile* infection in vivo (*p* < 0.05). This study demonstrates that bacteria-free supernatant is sufficient to protect mice from *C.difficile*; and could be further explored as a therapeutic strategy against *C.difficile* infection.

## Abstract (web summary)

An 18-member and a 4-member community consisting of commensal bacterial strains protected mice against *C. difficile* infection. The benefit of the 4-member community is mediated by secreted product(s) and not only by the bacteria, by proteolyzing clostridial toxins.

## Introduction

The gastrointestinal (GI) microbiota (GIM) plays a central role in human health, including immunomodulation^1,2^, influencing inflammation in the GI tract^3^ and resistance to pathogen colonization^4^. Antibiotics reduce the diversity of the GIM and have been shown to promote pathogen colonization, including colonization by *Clostridioides difficile* (CD)^4^. CD is the leading cause of hospital-associated diarrhea^5,6^ with an estimated annual incidence rate of 2.24 per 1000 admissions^7^. CD ribotypes 027 and 078 represent two of the most common ribotypes and have been linked to both severe and recurrent CD infection (CDI) as well as institutional CD outbreaks^8–10^.

CDI results in symptoms ranging from mild diarrhea to pseudomembranous colitis and toxic megacolon^11^. The standard of care for CDI is antibiotic therapy; however, nearly one in four individuals experience CDI recurrence within 30 days of completing treatment^12^, leading to increased morbidity and mortality in patients and a high burden on the health care system^13^. The high rates of treatment failure and the impact of CD recurrence on patient outcomes and the health care system have been the driving force behind the development of novel therapeutics to treat CDI.

The pathophysiology of CDI is mediated by the production of two toxins that are produced by CD; toxin A (TcdA) and toxin B (TcdB). Strains of CD that do not contain TcdA and TcdB genes, i.e., non-toxigenic strains, do not have the ability to cause clinical CDI. The central role of toxins in CD pathogenesis have made toxin production the target of antibacterial compounds like fidaxomicin, a macrolide family protein synthesis inhibitor, that inhibits toxin production in CD and is currently used in the treatment of CDI^14^. In addition to therapeutics that target toxin production, those that target perturbations to the GIM have proven to be highly efficacious including the use of fecal microbiota transplantation (FMT) as a currently approved second-line treatment for recurrent CDI (rCDI)^15–18^ and Rebyota^TM^, the first FDA approved fecal microbiota product for the treatment of rCDI in adults^19^, both providing promising therapeutic alternatives to patients that fail antibiotic therapy. However, the use of FMT is faced with several logistical challenges including standardization and dealing with donor-to-donor stool variability, difficulties with donor recruitment, intensive donor screening for established and emerging pathogens and high cost associated with delivering the product to patients^20–25^. Products like Rebyota^TM^ mitigate some of the variability and logistical challenges of FMTs, however the mechanism of action of both FMTs and therapies like Rebyota^TM^ remain unclear.

Previously, our group developed a defined microbial ecosystem therapeutic (MET-1) composed of 33 bacterial strains isolated in a laboratory environment from the feces of a healthy human volunteer, which protected mice and humans against CDI^26,27^. MET-1, in contrast to FMTs, is a well-defined bacterial community of commensal bacterial strains cultured in a controlled laboratory environment^26^. Although the minimum number of strains that are necessary for inclusion in this and similar formulations and their mechanisms of action remain unclear, the development of DMCs represents a promising strategy for treating patients with CDI. However, even with controlled laboratory environments and using previously characterized strains, there are still manufacturing challenges to consider including lot-to-lot variability, bacterial strain stability, and the potential risk of commensal bacteria causing illness, specifically in critically ill patients^28^.

Here we present data that demonstrates that a smaller community of bacteria including an 18-member community was able to protect against CD027 and CD078 ribotypes in a mouse model. The clinical and epidemiological significance of CD027 and CD078 led us to evaluate these two ribotypes to gain insights into strain-specific differences in pathogenicity and the activity of DMCs against different CD ribotypes.

We further refined the larger community, DMC-18, down to a 4-member community to identify potential mechanism(s) responsible for providing protection against CDI in vitro and in vivo. We demonstrate that the delivery of a bacteria-free conditioned media (CM) of the refined 4-member community (DMC-4) protected mice from CDI. DMC-4 CM neutralized CD toxin activity in vitro and in vivo, and acted by proteolyzing both TcdA and TcdB CD toxins to smaller, inactive fragments. These results suggest that a secreted bacterially-derived protease(s) may be acting on CD toxins directly, identifying a mechanism by which DMCs may be protecting against CDI; opening up the potential to develop bacteria-free CD therapeutics.

## Results

### Oral gavage of DMC-18 protected mice from CDI

**Fig. 1:**
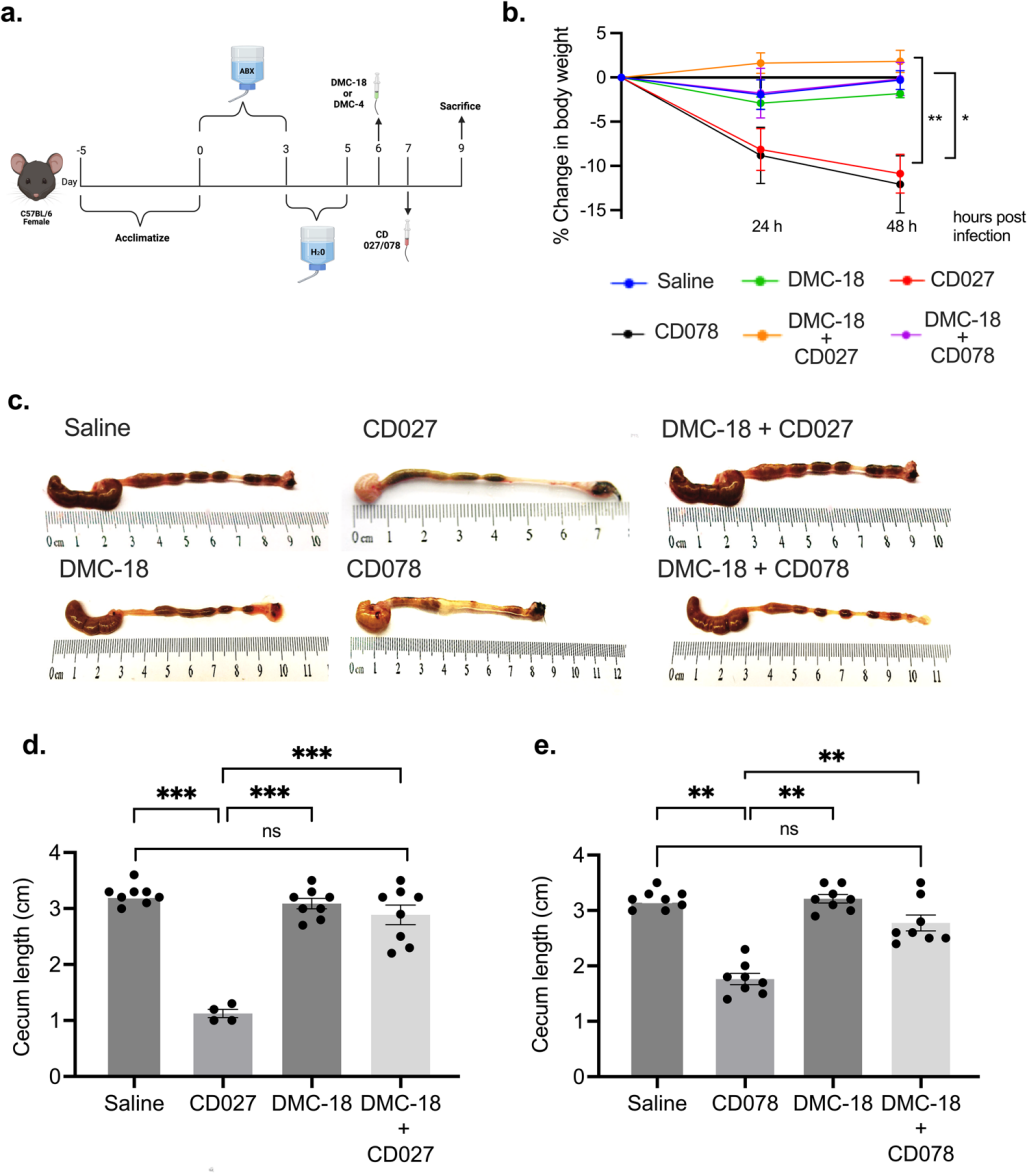
Pre-treatment with DMC-18 protected mice from CD-mediated weight loss and disease pathology. **a** Schematic of the CD mouse model used in this study. Mice were given an antibiotic cocktail *ad libitum* in their drinking water for three days, and then water for the next two days. On day 6, mice were gavaged 150 μl of thawed DMC-18 mixtures (prepared in the proportions described in Table 1), followed by CD027 or CD078 challenge (1 × 10^5^ CFU) on day 7. Mice were weighed, and their stools were collected daily. On day 9, mice were euthanatized by cervical dislocation. Blood, stool, colon, and cecum were harvested. Created with BioRender.com. **b** DMC-18 treated mice were protected from significant weight loss following CD027 or CD078 infection (two-way ANOVA with Tukey’s test, *n* = 8 for each group, *p < 0.05, **p < 0.01). **c** Representative images of the gross morphology of the ceca and colons from all groups of mice taken at the same magnification (20X). Uninfected mice and those exposed to DMC-18 prior to CDI had large ceca filled with feces and well-defined fecal pellets in the colon, while infected animals showed a reduction in their ceca size and demonstrated colitis. **d** Ceca were measured to compare sizes between uninfected, CD-infected and CD-infected+DMC-18 mice with CD027 and **e** CD078. Mice infected with CD had significant colonic shortening that was prevented when given DMC-18. A one-way ANOVA with Dunnett’s correction, *n* = 8 for each group was performed (***p < 0.001). Error bars represent standard error of the mean.

**Fig. 2:**
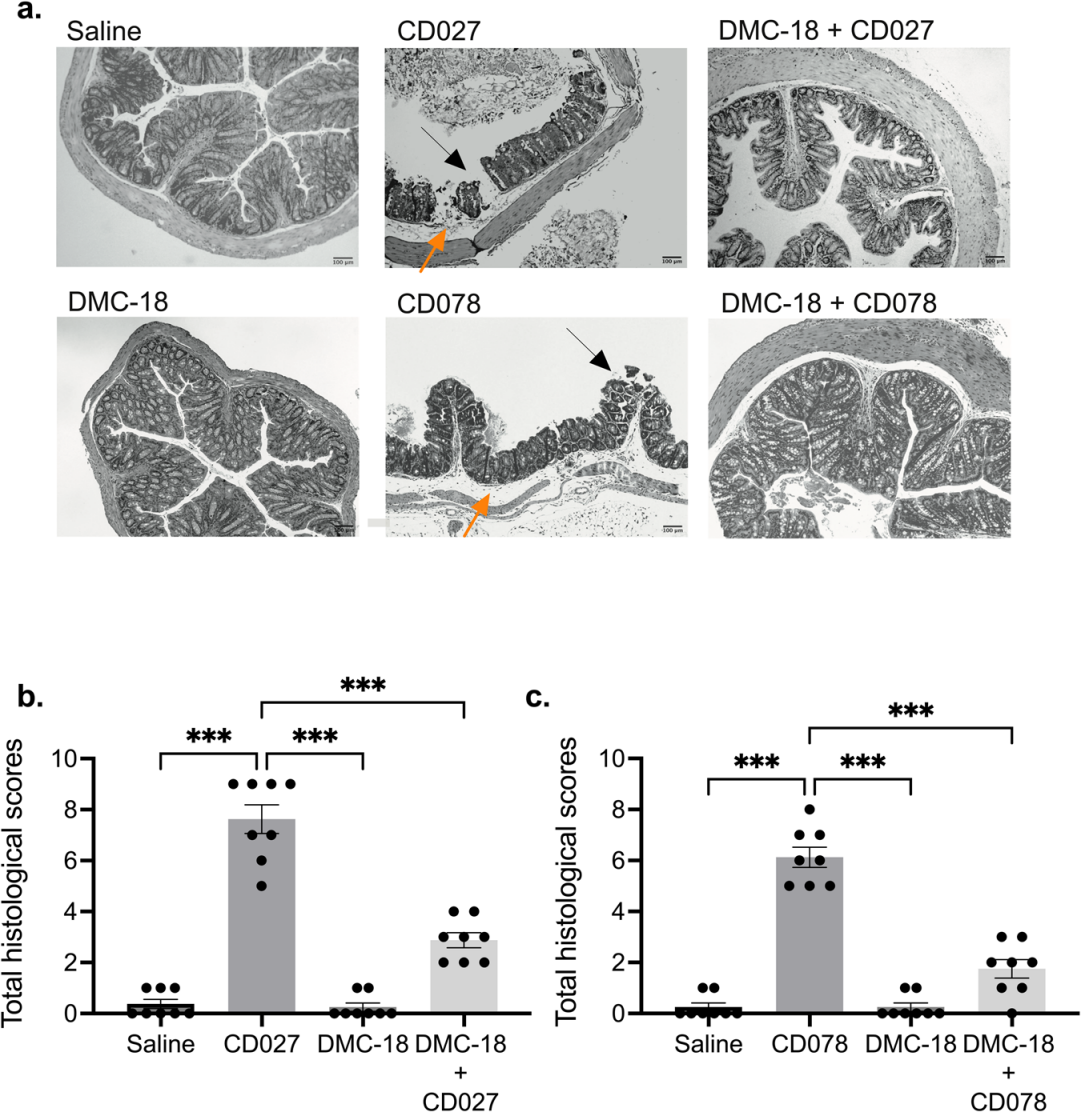
Histology of the murine colon revealed damage in CD-infected mice. **a** Representative H&E stained images of the colon of all mouse groups. Mice treated with CD027 and CD078 had evidence of increased epithelial injury (black arrow), mucosal edema (orange arrow) and neutrophil infiltration. Scale bar, 100 μm. Histological scores by two individuals in a blinded fashion performed on all groups enumerating the degree of edema, neutrophilic infiltration, and epithelial cell damage following CD027 (**b**) and CD078 infection (**c**). The scores ranged from 0 (no damage) to 3 (severe damage/fulminant disease), for a total of 9. DMC-18 exposure prior to CD infection reduced CD-mediated damage in mice infected with CD027 or CD078. CD infected mice had significantly higher scores compared to CD + DMC-18 mice (Kruskal–Wallis with Dunn’s test, *n* = 8 for each group, ***p < 0.001). Error bars represent standard error of the mean.

DMC-18 was administered to mice via oral gavage after three days of antibiotic exposure as per Martz et al. ^26^ (Fig. 1a). Mice were infected with either CD027 or CD078 ribotypes. Exposure to DMC-18 prior to CDI protected mice from CD-mediated weight loss at 24 h and 48 h after infection with CD027 (DMC-18 + CD027) and CD078 (DMC-18 + CD078) (Fig. 1b, *p* < 0.05, *p* < 0.01; respectively) compared to CD-infected controls (Fig. 1b). Overt signs of CDI in mice include hunched posture, reduced movement, bloody stool, and diarrhea. Post-mortem examination of murine colons demonstrated that mice infected with CD exhibited colonic inflammation and ceca shortening (Fig. 1c, middle panels, Fig. 1d and Fig. 1e, *p* < 0.01 for both). In contrast, DMC-18 + CD groups had intact stool pellets (Fig. 1c, right panels), experienced no ceca shortening (Fig. 1d, e, *p* = ns for both), no bloody stool, and did not display overt signs of illness, similar to the observations seen in uninfected mice. Histology of transverse colon sections revealed CDI-induced colitis, including epithelial necrosis (Fig. 2a, black arrows), cellular influx into the colonic mucosa (Fig. 2a), and severe submucosal edema in CD-infected mice (Fig. 2a; orange arrows) evident in mice infected with CD027 and CD078 (Fig. 2a). In contrast mice exposed to DMC-18 and infected with CD027 (Fig. 2a) and CD078 (Fig. 2a) had no evidence of CD-mediated epithelial necrosis, cellular influx or submucosal edema. Mice that received DMC-18 prior to CD had significantly lower histological scores (Fig. 2b, c, *p* < 0.001), although mice exposed to DMC-18 and infected with CD did have higher histological scores than uninfected controls (DMC and saline).

### DMC-18 treated mice had lower stool concentrations of TcdA and TcdB

**Fig. 3:**
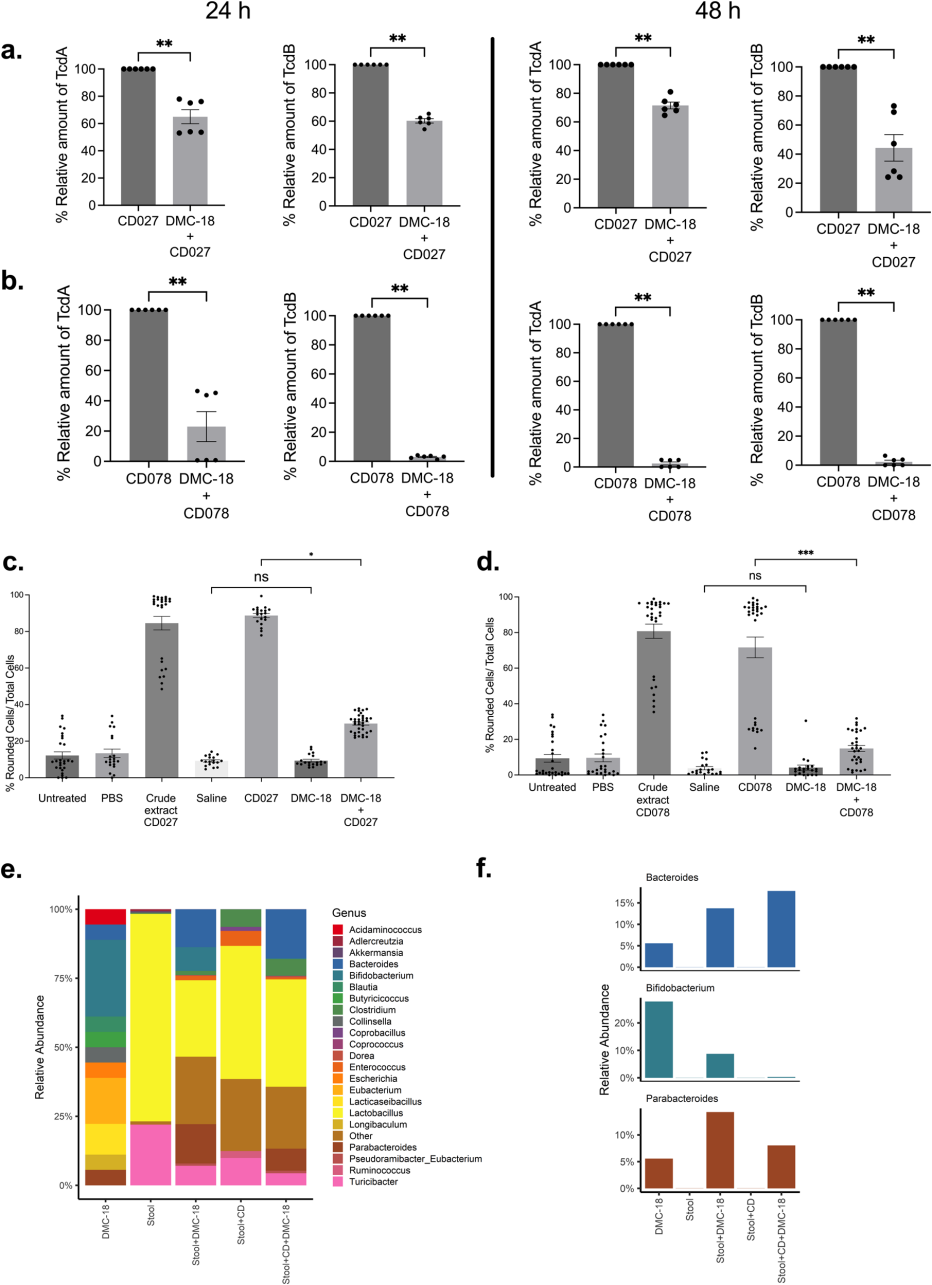
DMC-18 pre-treatment reduced toxin levels in mouse stool. CD + DMC-18 mice had significant reduction in relative concentrations of TcdA and TcdB in both CD027 (**a**) and CD078 (**b**) infected mice at 24 h and 48 h of infection (Mann–Whitney and a two-tailed test, *n* = 8 per group, **p < 0.01 for all groups tested in triplicate). Fibroblast cell rounding assays revealed that CD + DMC-18 mice had reduced toxin-mediated cytotoxicity in their fecal pellets compared to CD-mice infected with both CD027 **c** and CD078 **d** at 48 h post infection (Kruskal–Wallis with Dunn’s test was used to analyze the data, *n* = 8, **p < 0.01, ***p < 0.001). **e** 16S rRNA sequencing of murine stool from all groups demonstrated that DMC-18 led to a significant change in the relative abundance of bacteria in stool. **f** When comparing CD-infected mice treated with DMC-18 (Stool + CD + DMC-18) to CD-infected mice not exposed to DMC-18 we identified three bacterial genera only present in DMC-18, CD + DMC-18 but not CD-infected mice. *Bifidobacterium, Parabacteroides, and Bacteroides* were detected 24 h post-infection. These genera were absent prior to DMC-18 inoculation (stool) and in mice infected with CD but not treated with DMC-18 (Stool + CD). Operational taxonomic units were based on 97% identity; relative abundance is displayed. Error bars represent standard error of the mean.

CD toxins (TcdA and TcdB) measured by ELISA in mouse fecal pellets infected with CD027 and CD078 were detectable as early as 24 h post-infection and increased further at 48 h post-infection (*p* < 0.01 for all; Fig. 3a, b), in line with growth kinetics of CD previously reported in mice^29^. In contrast, DMC-18 + CD027 and DMC-18 + CD078 had significantly lower TcdA and TcdB in stool at both 24 h (Fig. 3a) and 48 h (Fig. 3a) post-infection (Fig. 3a, b, *p* < 0.01) suggesting that members in DMC-18 or their products may be interfering with TcdA and TcdB production or interfering with toxin activity in vivo.

To confirm if the decrease in TcdA and TcdB in mouse stool treated with DMC-18 following CD027 and CD078 infection were associated with reduced toxin activity, a fibroblast cell rounding assay previously used for the clinical diagnosis of CD was used to measure CD toxin activity^26,30–32^. Stool from DMC-18 + CD027 and DMC-18 + CD078 induced less cell rounding at both 24 h and 48 h compared to CD027 and CD078 infected mice (Fig. 3c, d, *p* < 0.001 for both and Supplementary Fig. 1). Incubation of stool from DMC-18 control group did not result in cell rounding and was comparable to cells exposed to PBS-buffer (Fig. 3d and Supplementary Fig. 1).

### 16S rRNA analysis of murine stool

Illumina 16S rRNA gene sequencing analysis on murine stool revealed that antibiotic exposure resulted in a dramatic shift in stool bacterial communities as early as 24 h post exposure (Fig. 3e). We next evaluated which of the DMC-18 members were found in mice post CD infection. Only three bacterial genera found in DMC-18 were found to be enriched and detectable in the stool of mice infected with CD and protected from CDI (Fig. 3f and Supplementary Figure 2). DMC-18-exposure in mice resulted in the enrichment of *Parabacteroides, Bacteroides*, *and Bifidobacterium* (Fig. 3f). We hypothesized that these 4 bacterial strains may represent keystone species providing protection from CDI. The remaining 14 strains, and 11 genera, from DMC-18 were not detectable in stool of the CD + DMC-18 group post-DMC-18 inoculation (Supplementary Figure 2). As expected, mice infected with CD had increasing relative abundance of CD (Supplementary Figure 2). There was no difference in CD relative abundance between CD + DMC-18 and CD-infected mice (Supplementary Figure 2). We therefore decided to create a refined community consisting of the 4 bacterial strains (called DMC-4) and evaluate its ability to neutralize CD toxin activity in vitro and protect mice from CDI in vivo.

### Oral gavage of DMC-4 protected mice from CDI

**Fig. 4:**
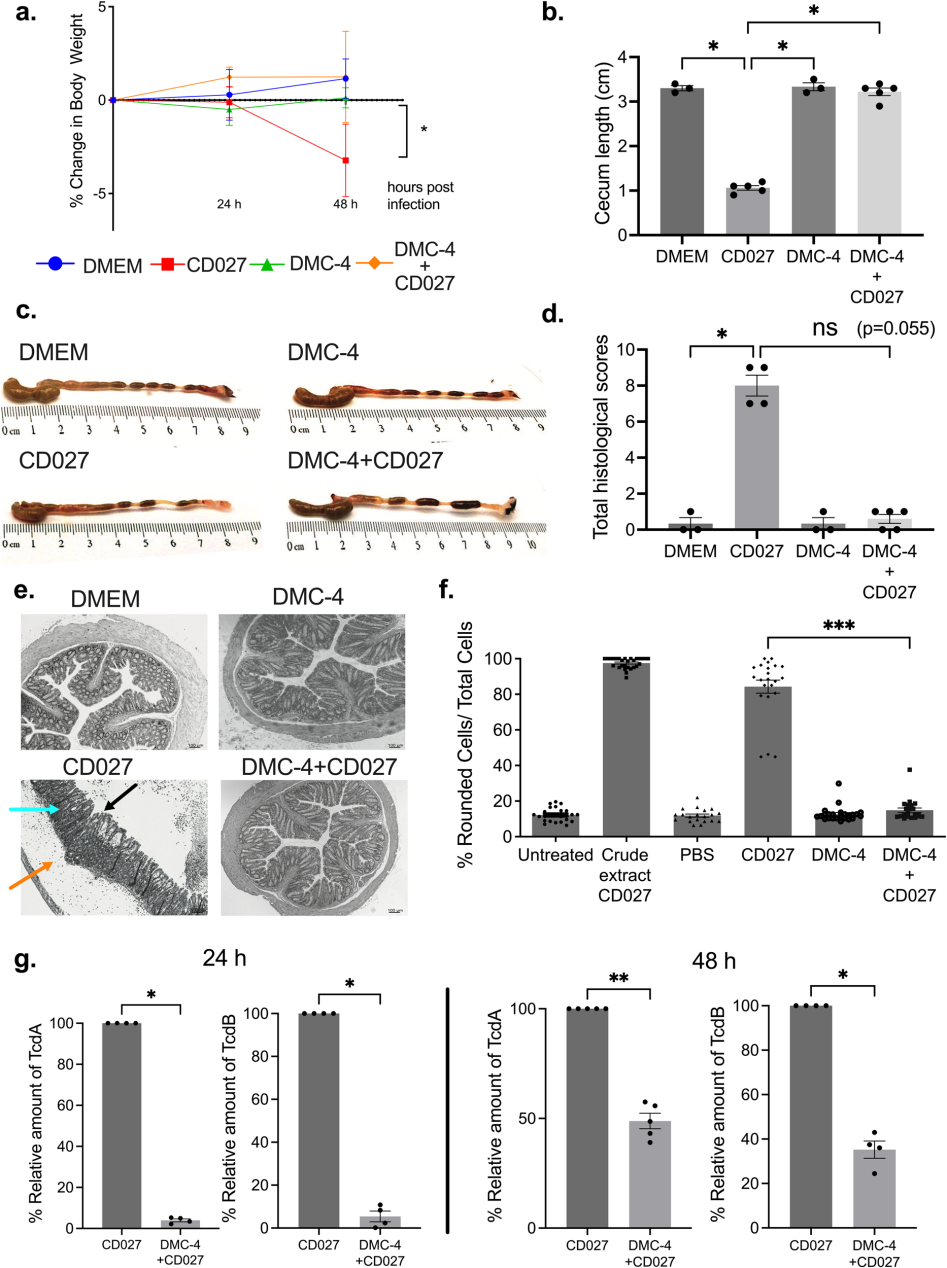
Pre-treatment with DMC-4 protected mice from CD-mediated weight loss, disease pathology and reduced toxin levels in stool. **a** Following the discovery that 4-members of DMC- 18 were found in the stool of mice protected from CDI, but not in the stool of CD-infected mice, a bacterial community of 4-bacteria (called DMC-4) was developed. DMC-4 treated mice were protected from significant weight loss following CD027 (two-way ANOVA with Tukey’s test, control animals *n* = 3 and infected animals *n* = 5, *p < 0.05). **b** Ceca from all mice were measured to compare sizes between uninfected, CD027-infected and DMC-4 + CD027 mice, similar to DMC-18; DMC-4 exposed mice were protected from ceca-shortening (one-way ANOVA with Dunnett’s correction, control animals *n* = 3 and infected animals *n* = 5, *p < 0.05, **p < 0.01). **c** Representative images of the gross morphology of the cecum and colon from all groups of mice taken at the same magnification (20X). **d** Histological scores of all treatment groups based on the degree of edema, neutrophilic infiltration, and epithelial cell damage following CD027. DMC-4 exposure prior to CD infection reduced CD-mediated damage in mice infected with CD027 (Kruskal–Wallis with Dunn’s test, control animals *n* = 3 and infected animals *n* = 5, *p < 0.05). **e** DMC-4 exposure reduces toxin activity and cytotoxicity of fecal pellets from CD027-infected mice Representative H&E stained images of the colon of all mouse groups demonstrated that CD027infected mice had evidence of increased epithelial injury (black arrow), mucosal edema (orange arrow) and neutrophil infiltration (blue arrow). Scale bar, 100 μm. **f** Cell rounding assays also demonstrated significantly lower toxin activity in stool from mice infected with CD027 + DMC-4 infected mice 48 h after the infection compared to the CD027 group (Kruskal-Wallis with Dunn’s test, ***p < 0.001). Pooled supernatants from stool pellets, control animals (*n* = 3) and infected animals (*n* = 5). Error bars represent standard error of the mean. **g** TcdA and TcdB quantifications in DMC-4-treated mice 24 and 48 h after CD027 infection were significantly lower than in CD-infected mice (*t*-Test with Mann–Whitney and two-tailed test, *p < 0.05. All groups were replicated in quadruplicate).

To evaluate the protective capacity of DMC-4 in vivo, mice were orally gavaged with DMC-4 prior to infection with CD027 according to the previously described protocol (Fig. 1a). DMC-4 exposed mice experienced reduced weight loss 48 h post-infection compared to untreated CD027 controls (Fig. 4a, *p* < 0.05). Mice treated with DMC-4 experienced less ceca shortening compared to CD027 mice (Fig. 4b, c, *p* < 0.05). Histological scoring of DMC-4 + CD027 mice performed in a blinded fashion enumerating epithelial injury, mucosa edema and neutrophil infiltration was numerically lower, but did not reach significance (Fig. 4d, e, *p* = 0.055). Fecal pellets isolated from mice exposed to DMC-4 had significantly lower CD toxin activity in vitro *at* 48 h post infection (Fig. 4f, *p* < 0.001 and Supplementary Fig. 3) and lower relative concentrations of TcdA and TcdB toxins at both 24 h and 48 h post infection (Fig. 4g; *p* < 0.05 for both time points).

### CM from DMC-4 neutralized CD toxin in vitro and prevents Glucosylation of Rac1

**Fig. 5:**
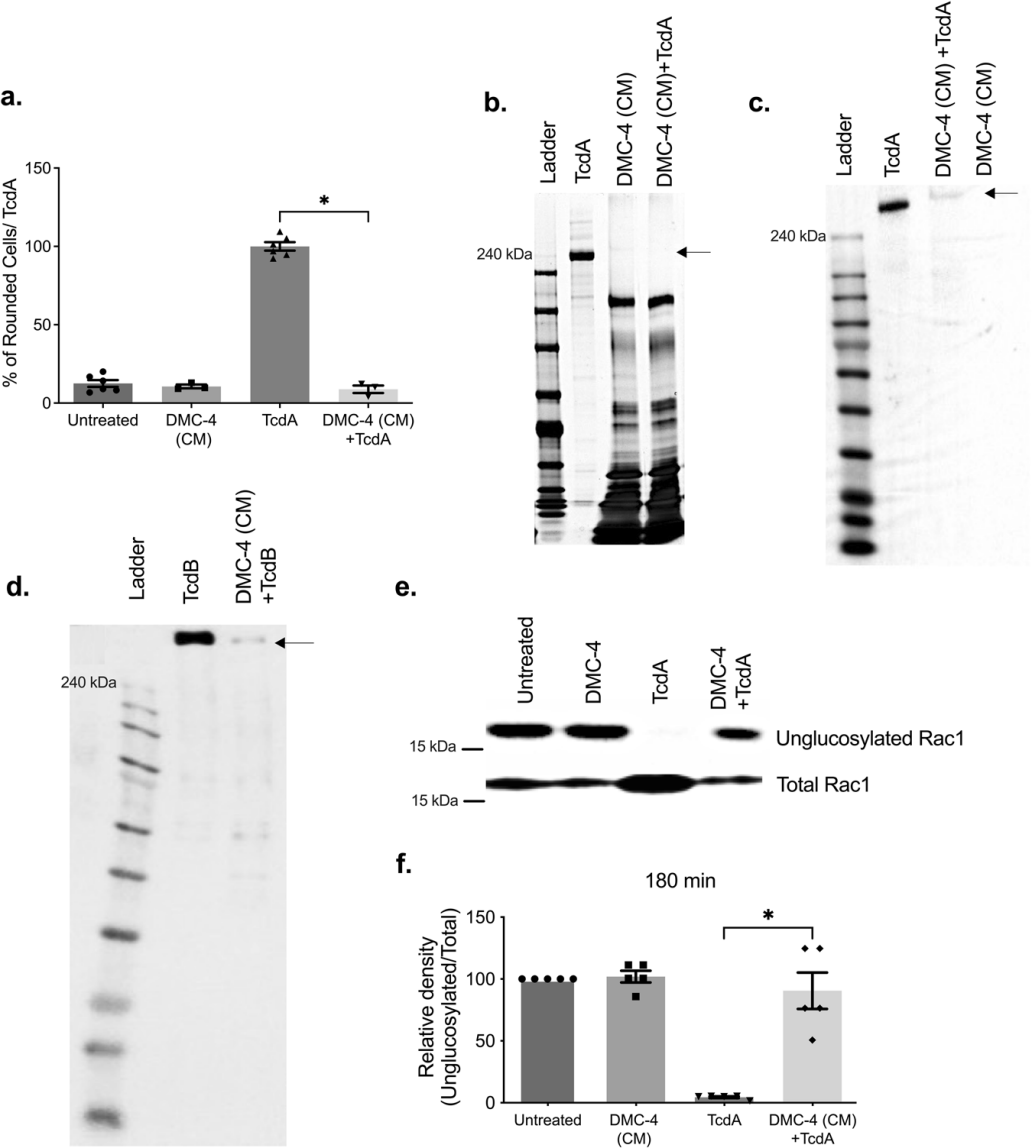
DMC-4 (CM) proteolyzed TcdA and TcdB and prevented glucosylation of Rac1. **a** In addition to DMC-4 inhibiting toxin activity; conditioned media (CM) from DMC-4 also inhibited TcdA-mediated cell rounding in vitro (pooled results of assays run in triplicate, Kruskal-Wallis with Dunn’s test, *p* < 0.05). Silver staining (**b**) and western blot demonstrated the loss of purified TcdA (100 ng) (**c**) and TcdB (100 ng) (**d**) when incubated with DMC-4 (CM) for 1h. **e** Densitometry analysis was conducted to quantify glucosylation activity of Rac1 using DMC-4 (CM). Toxin-mediated glucosylation was inhibited (**e**, **f**) in cells treated with DMC-4 (CM) supernatants (*p* < 0,05, One-way ANOVA with Dunnett’s test. Error bars represent standard error of the mean).

Next, to elucidate a potential mechanism of action behind the efficacy of DMC-4, we used the filtered supernatant of spent bacterial CM to examine if protection against CDI was mediated by live bacteria or secreted bacterial products. Co-incubation of DMC-4 conditioned media (DMC-4 CM) with TcdA almost completely abrogated TcdA activity and protected cells from toxin-mediated cell rounding (Fig. 5a, *p* < 0.05). A silver stain (Fig. 5b) and western blot (Fig. 5c) analysis confirmed that DMC-4 CM incubated with TcdA resulted in the degradation of TcdA to smaller fragments within 2 h of incubation. The same phenomenon was observed when a western blot of TcdB found that DMC-4 CM led to the loss of parent TcdB in vitro (Fig. 5d). We next investigated if DMC-4 was able to inhibit Rac1 glucosylation, a Rho subfamily protein that are substrates for CD toxins^33^.

Rac1 glucosylation by TcdA was prevented when cells were incubated with DMC-4 CM. NIH 3T3 cells treated with DMC-4 CM and TcdA had higher levels of unglucosylated Rac1 (relative density of 90.57) comparable to untreated or DMC-4 cells, while the TcdA treated positive control was almost absent (relative density of 4.46, Fig. 5e, f, *p* < 0.05).

### CM from DMC-4 protects mice from CDI

**Fig. 6:**
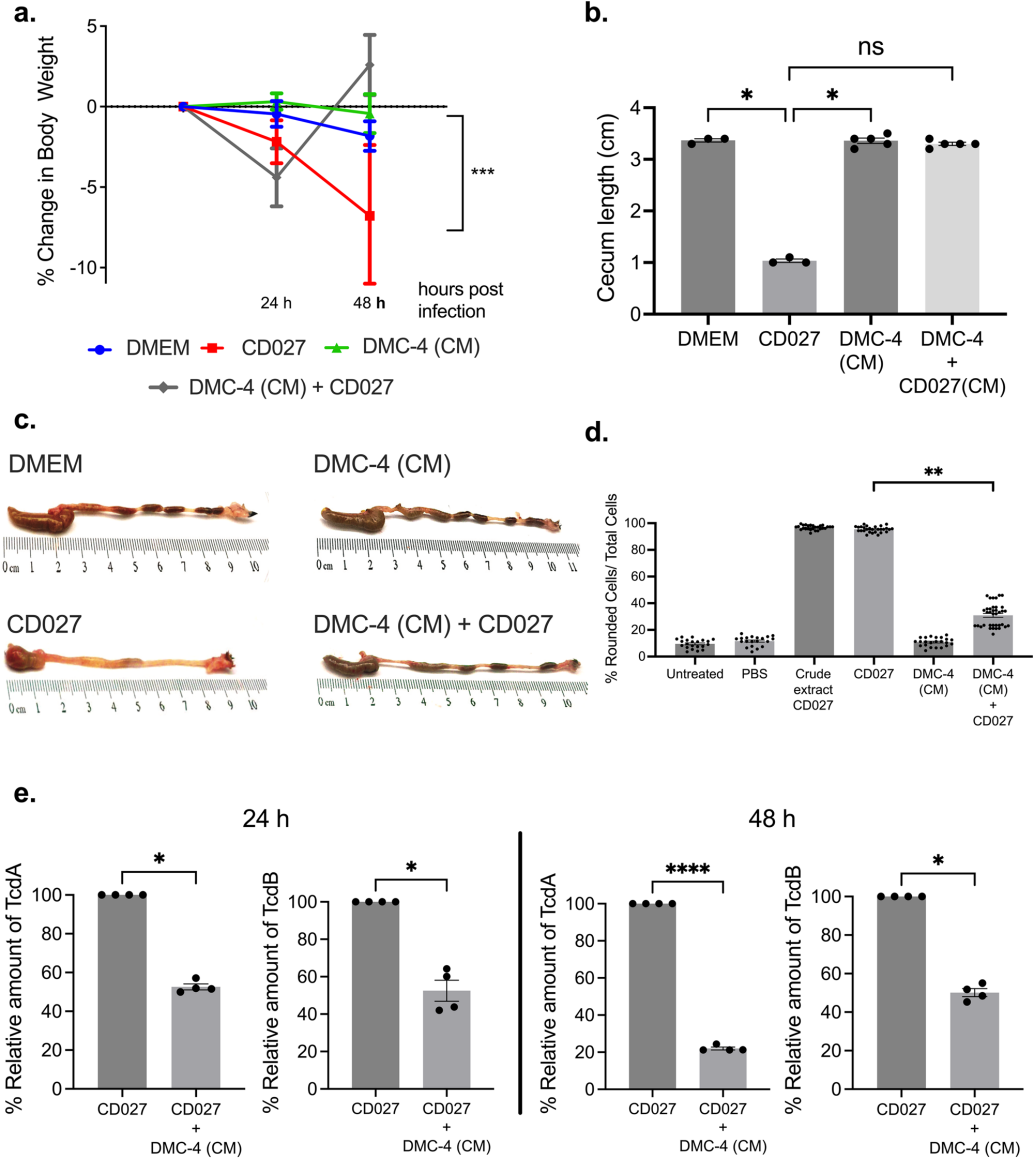
Pre-treatment with DMC-4 (CM) protected mice from CDI. **a** DMC-4 (CM) treated mice were protected from significant weight loss following CD027 infection compared to CD-infected mice (two-way ANOVA with Tukey’s test, DMEM *n* = 3, CD027 *n* = 4, DMC-4 (CM) *n* = 4 and DMC-4 (CM) + CD027 *n* = 4, *p < 0.05). **b** DMC-4 CM + CD mice were protected from ceca shortening compared to CD-infected mice (one-way ANOVA with Dunnett’s correction, DMEM *n* = 3, CD027 *n* = 4, DMC-4 (CM) *n* = 4 and DMC-4 (CM) + CD027 *n* = 4, *p < 0.05). **c** Representative images of the gross morphology of the ceca and colons from all groups of mice taken at the same magnification (×20) demonstrated that uninfected mice and those exposed to DMC-4 (CM) prior to CD infection had large ceca filled with feces and fecal pellets in the colon, while CD infected mice had reduced cecum size and morphological evidence of colitis. **d** Stool from mice infected with CD027 resulted in significantly higher toxin-mediated cell rounding compared to DMC-4 CM + CD027 (Kruskal-Wallis with Dunn’s test, **p < 0.01). Pooled supernatants from stool pellets, control animals *n* = 3 and infected animals *n* = 5. **e** TcdA and TcdB quantifications in DMC-4 (CM) treated mice after CD027 infection demonstrated that CD + DMC-4 CM mice had significantly lower levels of TcdA and TcdB at both 24 h and 48 h post-infection (Mann–Whitney test was used to analyze the data (*p < 0.05). All groups were carried out in triplicate). Error bars represent standard error of the mean.

Finally, we investigated if DMC-4 CM can protect mice against CDI according to the experimental protocol and timeline outlined in Fig. 1a. Mice pre-treated with DMC-4 (CM) were significantly protected from weight loss 48 h post CD027 infection (Fig. 6a, *p* < 0.05). DMC-4 (CM) + 027 mice displayed longer colons, with intact fecal pellets compared to CD027 mice and their ceca were significantly longer (Fig. 6b, c) and cell rounding assays revealed that stool from DMC-4 (CM) + 027 mice rounded significantly less cells 48 h post infection compared to stool from CD027 mice, suggesting a loss in toxin activity when mice are pre-treated with DMC-4 CM (Fig. 6d, *p* < 0.001). In addition, relative amount of TcdA and TcdB at 24 and 48 h post infection was significantly lower in DMC-4 (CM) + 027 mice compared to CD027 mice (Fig. 6e, *p* < 0.05).

## Discussion

Toxins are potent virulence factors that allow pathogens to circumvent host immune responses including suppression of immune cells, inducing cellular apoptosis, acting as super-antigens, promoting phagocytosis escape, and promoting pathogen germination^34,35^. The pathophysiology of CD is mediated by two toxins as previously described, TcdA and TcdB, that are encoded by a pathogenicity locus^36^. The central role of toxins in this disease has led to the development of several therapies specifically targeting or neutralizing CD toxins including monoclonal antibodies^37^, toxin binding agents^38^, small-molecule inhibitors^39^, narrow spectrum antibiotics^14^, supplementation with short-chain fatty acids, immunization^40^ and bacteriotherapy^41^. While bacteriotherapy is a promising approach, the mechanism(s) behind the efficacy of DMCs is not well understood. The goal of our study was to develop and characterize a defined bacteriotherapy to combat CDI and to help elucidate potential mechanisms behind the efficacy of DMCs. While DMC-18 protected mice from CDI, the 18-member community was too large and complex to enable our team to narrow down potential mechanisms of action. However, our results suggested that bacterial-free CM from both DMC-18 and a much smaller 4-member community was sufficient to protect mice from CD pathology shedding light on the mechanism of action behind DMC-4 protection. This is particularly exciting since the use of live bacterial biotherapeutics has inherent risks, including the potential to cause illness in patients with increased gastrointestinal permeability and immunosuppression, as is observed in CD patients^28^.

In this report, we provide evidence for the presence of a cell-free soluble bacterial product(s), produced by a 4-member DMC, capable of neutralizing CD toxin activity in vitro and protecting mice from CDI. The proteolysis of both toxins suggests that members of DMC-4 produce one or several bacterial protease(s) that are able to degrade CD toxins and neutralize their effects in vitro and in vivo. Isolation and further characterization of bacterial protease(s) present an important line of therapeutic strategies that can be used to combat infectious diseases in light of the emergence of multidrug resistance in toxin-producing bacteria including CD and *Shigella*^42,43^. The use of toxin-targeting proteases has previously been suggested and there is evidence that *Sacchromyces boulardii*^44^ harbours a 54 kDa protease that proteolyzes TcdA and prevents CD pathogenesis^44^, but no studies have identified bacterially derived soluble factors capable of proteolyzing both *TcdA* and *TcdB*, and protecting mice from the development of CDI.

Several families of bacterial proteases including caseinolytic proteases, FtsH, Lon, Hs1UV, and high temperature requirement A have been studied and are important for modulating bacterial growth, protein quality control, homeostasis, regulating prokaryotic division, host-cell invasion, lipopolysaccharide biosynthesis; serving to ensure bacterial survival and the biosynthesis of key virulence factors^45–47^. The role of bacterial proteases in bacterial homeostasis and virulence has led several groups to suggest that targeting bacterial proteases may be promising therapeutic strategy^45,48^.

Further studies need to focus on investigating if the products responsible for CD toxin neutralization are produced by an individual, or multiple member of DMC-4 to further efforts to understand/identify the mechanisms required for CD toxin neutralization. In addition the impact of these soluble factors on CD colonization and spore formation needs to be evaluated since preliminary data from this study suggests that DMC-18 did not impact CD colonization since relative abundance of the genera *Clostridium* (now *Clostridioides*) were similar in mice infected with CD or DMC-18 + CD (Supplementary Figure 2). Although exciting, additional work characterizing, isolating, and determining the specificity of CD-active proteolytic compounds needs to be undertaken to ensure that this form of therapy is safe and specific for use. In addition, our animal model only investigates the efficacy of DMC-4 CM prophylactically because mice in this model develop rapid and aggressive CDI precluding our ability to measure the activity of DMC-4 CM as a therapeutic.

The results of this study provide exciting preliminary evidence that bacterial-free CM alone from a refined DMC can prophylactically protect against CDI via a previously unknown mechanism of action^49^. Further studies in our laboratory are currently underway to isolate and characterize the product(s) with anti-CD toxin activity.

## Methods

### Ethics statement

This study was carried out in accordance with the Canadian Council of Animal Care guidelines and approved by the Queen’s University Animal Care Committee and the Biosafety committee.

### Development of the DMC-18 microbial community

We developed a defined community of 18 bacterial isolates, some of which are also members of MET-1^26^, that remained stable after freezing procedures (Table 1) based on the ability to culture each isolate post-freezing. In brief, each isolate was cultured from frozen stocks on fastidious anaerobic agar (Neogen, UK), supplemented with 5% defibrinated sheep blood (Cedarlane, Canada) in an anaerobic chamber (Ruskinn Bugbox, UK) at 37 °C for 24 h. Bacterial colonies were used to inoculate 15 mL of broth medium specific for each strain (Table 1), which was then incubated anaerobically at 37 °C for 24 h. Individual cultures were centrifuged for 10 min at 2704 x *g*, and the supernatants were discarded. Bacterial cell pellets were suspended in 15 mL of 0.9% NaCl, and the DMC-18 mixtures were prepared in predetermined proportions (Table 1). DMC-18 aliquots were then frozen at −80 °C for at least 24 h.

**Table 1:** Constituents of DMC-18.

Closest species^a^ (% Identity)	Broth media requirements^b^	Volume of culture per Frozen DMC-18 aliquot
*Acidaminococcus intestini* (100%)	WCB	1 mL
***Bacteroides ovatus*** **(99%)**	**WCB** + **G** + **SA** + **SS**	**1** **mL**
*Bifidobacterium faecale* (100%)	WCB + G + SA + SS	500 μL
***Bifidobacterium longum*** **(99%)**	**WCB** + **G** + **SA** + **SS**	**500** μ**L**
***Bifidobacterium longum*** **(99%)**	**WCB** + **G** + **SA** + **SS**	**1** **mL**
*Bifidobacterium pseudocatenulatum (99%)*	WCB + G + SA + SS	1 mL
*Bifidobacterium stercoris* (98%)	WCB + G + SA + SS	1 mL
*Blautia luti* (95%)	WCB	1 mL
*Butyricicoccus faecihominis* (98%)	WCB + G + SA + SS	1 mL
*Collinsella aerofaciens* (99%)	WCB	500 μL
*Escherichia fergusonii* (100%)	WCB	500 uL
*Eubacterium callanderi* (97%)	WCB	500 μL
*Eubacterium fissicatena* (99%)	WCB + G + SA + SS	1 mL
*Eubacterium ventriosum* (99%)	WCB + G + SA + SS	1 mL
*Lacticaseibacillus casei* (100%)	WCB + L	500 μL
*Lacticaseibacillus paracasei* (100%)	WCB + L	500 μL
*Longibaculum muris* (93%)	WCB	1 mL
***Parabacteroides distasonis*** **(99%)**	**WCB** + **G**	**500** μ**L**

Broth media and volumes required to prepare the frozen formulations.

*WCB* Wilkins Chalgren Broth (33 g/L), *G* Glucose (3 g/L), *SA* Sodium acetate (2 g/L), *SS* Soluble starch (4 g/L), *L* Lactose (4 g/L).

^a^Closest species match was inferred by alignment of the full-length 16S rRNA gene sequence to the NCBI database. Some strains were identified as belonging to the same species by full-length 16S rRNA gene sequence alignment, but we consider them as different strains due to differences in colony morphology, antibiotic resistance patterns and growth rates.

^b^Bolded organisms were incorporated into DMC-4.

### Growth of CD in culture

Clinically toxigenic CD ribotypes 027 and 078 (CD027, CD078) were isolated from stool samples of confirmed clinical cases with CDI at Kingston Health Sciences Center and cultured on Cycloserine Cefoxitin Fructose Agar, under anaerobic conditions (90% N_2_, 5% CO_2_, and 5% H_2_) at 37 °C for 48h^26^. After incubation, a single colony was used to inoculate 5 mL of Brain Heart Infusion broth (Difco Laboratories, France). For mouse infections, CD was grown anaerobically at 37 °C for 24 h. Mice were gavaged with 1 X 10^5^ CFU of CD027 or CD078 vegetative cells in brain heart infusion broth or just brain heart infusion in controls.

### CDI in mice pre-treated with DMC-18

The frozen DMC-18 formulation and DMC-4 were tested in vivo using an antibiotic CDI mouse model^50^. Six-week-old C57BL/6 female mice from Jackson Laboratory (USA) were acclimated to the animal facility for five days. Mice were given an antibiotic cocktail *ad libitum* in drinking water consisting of kanamycin 0.4 mg/mL (Sigma, Israel), gentamicin 0.035 mg/mL (Amresco, USA), colistin 850 U/mL (Sigma, China), metronidazole 0.215 mg/mL (Sigma, China) and vancomycin 0.045 mg/mL (Sigma, Israel), for three days. On day 6, mice were either administered (1) Dulbeccoʼs Modified Eagleʼs Medium (0.9% NaCl, 150 μL) or (2) DMC-18 (thawed culture, 150 μL) via oral gavage. On day 7, mice were infected with either CD027 or CD078 (fresh culture, 100 μL each) (Fig. 1).

To assess DMC-4 (CM) efficacy, we repeated the previous model but administered DMC-4 -CM via enema. On days 6, 7, and 8, mice were given DMC-4 (150 μl), which was grown in Dulbeccoʼs Modified Eagleʼs Medium (Sigma Aldrich, USA) at 37 °C for 72 h in anaerobic chamber (5% CO_2_, 10% Hydrogen and balance Nitrogen) and filtered through 0.22-micron filter. Mice not receiving DMC-4 (CM), received a vehicle control by enema. Then, mice were orally gavaged with either CD027 (1x10^5 CFUs in 150 μl) or vehicle control (brain heart infusion broth). Body weight was monitored daily, and stool samples were collected after each treatment set. On day 9, mice were euthanized by cervical dislocation in accordance with animal care protocols. Intestinal tissues were collected and placed either formalin-fixed or frozen at -80 °C. The collected sera and stool samples were stored at -80 °C for further use. Ceca tissues were also removed and fixed in 10% buffered formalin phosphate (Fisher Scientific, USA).

### Histology

Previously fixed ceca tissues were processed with ethanol, xylene and paraffin. Embedded tissues were sectioned 4-μm thick and stained with hematoxylin and eosin (H&E, Thermo Fisher Scientific, USA). An established graded scoring system was used to examine stained sections^50,51^. The scoring system accounts for 1) neutrophil migration and tissue infiltration, 2) hemorrhagic congestion, and 3) mucosal edema and epithelial cell damage. Each parameter was assigned a score between 0 and 3; a score of 0 indicates no pathologic damage, 1 mild, 2 moderate, and 3 total damage in the tissue; the sum of the scores represents the total damage in the tissue. Representative images of the tissues were captured using a microscope (Olympus BX71, USA) with a digital camera (Q-imagining Retiga-2000RV, USA).

### Quantification of stool CD toxin

TcdA and TcdB levels in mouse stool were quantified using an ELISA kit (tgcBIOMICS, Germany) according to the manufacturer’s instructions. In brief, 50 mg of mouse stool was homogenized in an Eppendorf tube with 450 μL of the kit dilution buffer and centrifuged at 2500 x *g* for 20 min. The pellet was discarded, and 100 μL of the supernatant was added to anti-TcdA/TcdB pre-coated wells and incubated for 1 h at room temperature. The plates were then washed (x3) with wash buffer and incubated with toxin-specific secondary antibodies (either anti-TcdA-HRP or TcdB-HRP conjugate) for 30 min. After washing the plate (x3), 100 μL of the substrate was added and incubated for 15 min at room temperature before adding 50 μL of stop solution. A microplate reader (Bio-Tek mQuant MQX200) was used to measure absorbance at 450 and 620 nm. A standard curve was generated using TcdA and TcdB pure toxins included in the kit to calculate sample concentrations. All samples were tested in triplicate.

### CD toxin cytotoxicity cell rounding assay

NIH 3T3 fibroblasts (ATCC) were grown in Dulbeccoʼs Modified Eagleʼs Medium media supplemented with 10% fetal calf serum (GIBCO, ThermoFisher Scientific, USA). Cells were seeded in 24-well flat-bottom tissue culture plates (Corning, USA) and incubated for 48-72 h to 70% confluency at 37 °C in an incubator supplied with 5% CO_2_. To evaluate the cytotoxicity of CD toxins in stool, 50 mg of murine stool was homogenized in 500 μL of PBS (NaCl, Sigma, USA; KCl, Bioshop, Canada; Na_2_HPO_4_, Bioshop, Canada; KH_2_PO_4_, Bioshop, Canada). After 30 min of centrifugation at 16,000 x *g*, supernatants were recovered. Fibroblasts were incubated for 2 h at 37 °C with 5% CO_2_ with 50 μL of fresh supernatants. Following incubation, all wells were washed with PBS and the cells were fixed for 30 min in 10% phosphate-buffered formalin. The cells were washed with PBS (2x) and stained with Giemsa (Sigma Aldrich, USA). Stains were incubated overnight and then washed out with PBS. Cells were imaged using a microscope (Olympus BX71) at 10X with a digital camera (Retinga-2000RV). Image J 1.51a software was used to count the cells (NIH, USA).

### 16S rRNA gene sequencing

DNA was extracted from 50 mg of frozen stool pellets using the DNeasy PowerSoil Pro Kit (Qiagen, USA), following the manufacturer’s instructions. 16S rRNA genes were amplified and barcoded using the Nextera DNA Library Preparation Kit (Illumina, FC-1211011, CA, USA). Briefly, 1 μL of extracted DNA was amplified using region of interest–

specific primers with overhang adapters. Forward:

5’TCGTCGGCAGCGTCAGATGTGTATAAGAGACAGCCTACGGGNGGCWGCAG-3’,

Reverse:5’GTCTCGTGGGCTCGGAGATGTGTATAAGAGACAGGACTACHVGGGTAT

CTAATCC-3) for the 16 S V3 and V4 regions, in a PCR reaction with Invitrogen Platinum™

Taq DNA Polymerase (Illumina, CA, USA). Illumina flow cell adapter sequences and a 12-bp barcode were then incorporated into the PCR primers, resulting in a fully Illumina-compatible sequencing library. DNA amplicon purity and concentration were quantified on a 2100 BioAnalyzer (Agilent Technologies, USA) and Qubit 3.0 Fluorometer (Thermo Fisher Scientific, USA).

Raw sequencing read quality was assessed using the tool FastQC v0.11.9. Low-quality nucleotides and adapter sequences were removed using Cutadapt v3.4 within the TrimGalore v0.6.6 wrapper. 16 S rRNA analysis and classification were performed using the Quantitative Insights into Microbial Ecology (QIIME 2 2019.7.0) software package^52^. Reads were processed with DADA2^53^ for quality filtering (forward and reverse reads were trimmed at 270 and 245 bases, respectively), denoising, and chimera removal. All sequencing reads were inserted into a reference phylogenetic tree using SATé-enabled phylogenetic placement and taxonomy was assigned to each read using the classify‐sklearn machine learning classifier^54^ against the Greengenes 13.8 99% operational taxonomic units reference sequences^55^. The taxonomy table was imported into R version 4.2.1 (R Project for Statistical Computing) for visualization using ggplot2 version 3.4.0 and RColorBrewer version1.1-3. Microbiome 16S rRNA sequencing data generated in this study have been deposited in the Sequence Read Archive under accession code PRJNA1029629.

### Rac1 quantification

DMC-4 (35 μL) was co-incubated with TcdA (1 μg) for 1 h at 37 °C and then added to NIH 3T3 cells monolayers for 3 h at 37 °C. Cells were harvested and lysed. Cell lysates (25 μL) were loaded into SDS-PAGE (15%). Western blot was performed to detect both unglucosylated Rac1(clone 102) and Rac1 total (clone 23A8). Primary antibodies (mouse monoclonal, BD Transduction Laboratories, USA, CAT:610650 for clone 102 and EMD Millipore, Germany, CAT: 05-389 for clone 23A8) were used at 1:2500 dilution and secondary (goat anti-mouse HRP, Invitrogen, USA) at 1:15000 dilution. Cells left untreated and exposed to DMC-4 served as controls. The ratio of unglucosylated to total Rac1 levels was calculated by densitometry using three repetitions of the experiments. The densitometry was done by using the BioRad Image Lab 6.1.

### Silver staining and western blot analysis

TcdA (1 μg) (List Biological Laboratories, Inc. California, USA) was incubated with DMC-4 (35 μl) for 1 h at 37 °C. Samples were loaded using 6X SDS loading buffer and heated at 95 °C for 10 min. All samples were loaded on to gradient SDS-PAGE gels (4-15%) after boiling. Silver staining was performed using the Pierce Silver Stain Kit as per the manufacturer’s instructions (Thermo Fisher Scientific. USA).

TcdA and TcdB (100 ng) were incubated with DMC-4 (35 μL) for 1 h at 37 °C. Samples were loaded using 6X SDS loading buffer and heated at 95 °C for 10 min. All samples were loaded into gradient SDS-PAGE gels (4-15%) after boiling. The polyvinylidene difluoride membrane (EMD Millipore, Germany) was incubated with either TcdA mouse monoclonal antibody (1:2500, Santa Cruz Biotechnology, Inc., USA, PCG4) or TcdB sheep polyclonal antibody (1:1,000 dilution, R&D Systems, USA, AF6246) overnight at 4 °C. After, the membrane was incubated with secondary antibody (1:20,000 dilution, goat anti-mouse HRP, Invitrogen, USA, CAT: 31430 for TcdA and 1:10,000, Donkey anti-Sheep HRP, Sigma-Aldrich, MO, USA, CAT: A3415 for TcdB) for 1 h at room temperature. Imaging of the membrane was performed using a Bio-Rad gel documentation system equipped with the Image Lab software^26^ (BioRad Laboratories Ltd., Canada).

### Statistics and reproducibility

Data were analyzed using GraphPad Prism Version 9.5 for Macintosh, GraphPad Software (USA). The results are expressed as the mean ± of the standard error of the mean (SEM). Eight mice were used per group (*n* = 8). Data were analyzed using a *t*-Test with Mann-Whitney, two-tailed test. Ordinary one-way analysis of variance (ANOVA) with Dunnett’s or Tukey’s test was performed to compare the difference between the means of more than two groups. A two-way ANOVA with Tukey’s test was performed to analyze the interrelationship of two independent variables. *T*-test with Kruskal-Wallis and Dunn’s test. *p* < 0.05 was chosen to reject the null hypothesis.

### Reporting summary

Further information on research design is available in the Nature Portfolio Reporting Summary linked to this article.

### Supplementary information

Supplementary Information

Description of Additional Supplementary Files

Supplementary Data 1

Reporting Summary

## Data Availability

Sequencing files for 16s rRNA are deposited in NCBI under accession code PRJNA1029629, biosample accession numbers SAMN38050861, SAMN38050862, SAMN38050863, SAMN38050864. Whole genome sequencing data for DMC-4 also available under PRJNA1029629. Unique data IDs are: *Bacteroides ovatus* (SAMN37875513 JAWUDM000000000), *Bifidobacterium longum* (SAMN37875515 JAWUDK000000000 and SAMN37875514 JAWUDL000000000), *Parabacteroides distasonis* (SAMN37875516 JAWUDJ000000000). Uncropped gels for Fig. 5b–e can be found under Supplementary Information as Supplementary Figs. 4–7 respectively. All other source data for this paper can be obtained in Supplementary Data 1.
