# Supplementary material for: Defined microbial communities and their soluble products protect mice from Clostridioides difficile infection
Source: Commun Biol. 2024 Jan 27;7:135. doi: 10.1038/s42003-024-05778-6 (PMC10821944; doi:10.1038/s42003-024-05778-6)
Supplement: Supplementary file 1 — Supplementary Information [file 42003_2024_5778_MOESM1_ESM.pdf]

Supplementary Figures

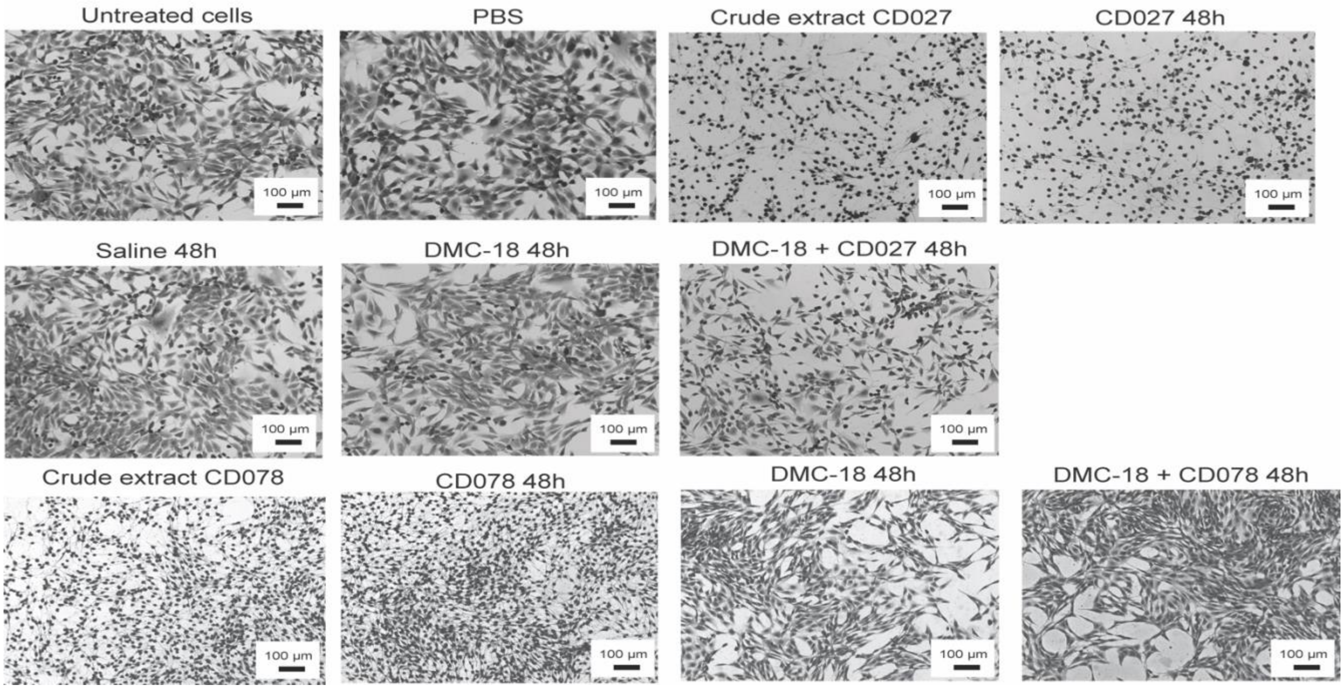

Supplementary Figure 1: Representative images of fibroblasts cultures stained with Giemsa that were incubated with stool extract from mice pre-exposed to DMC-18 and infected with CD027 or CD078. All experiments were performed using CD027 and CD078 crude extracts and stool extracts from CD027-infected mice as positive controls; PBS, DMC-18 stool extracts asnegative controls and untreated cells as reference.

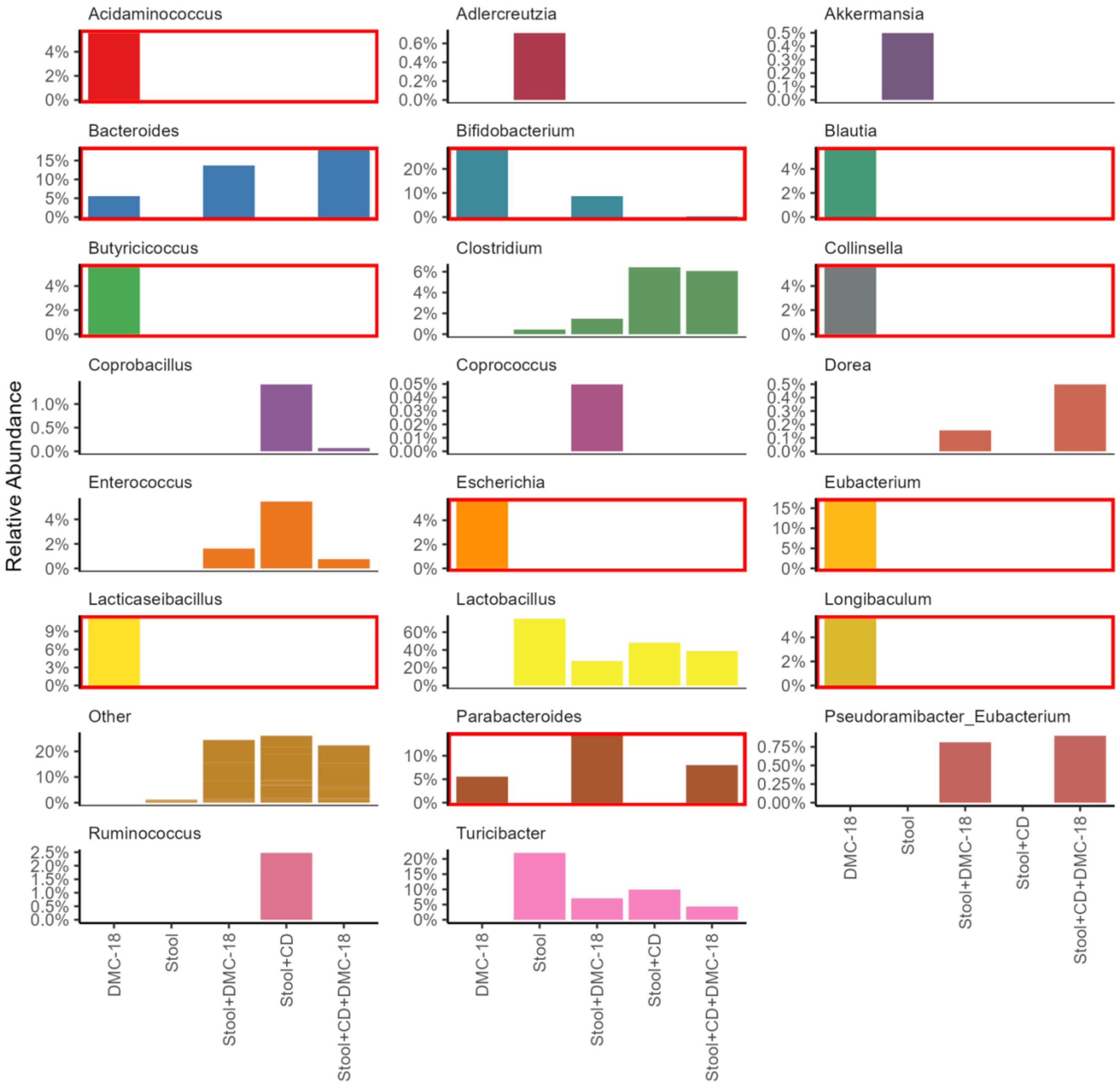

Supplementary Figure 2: 16s rRNA sequencing of individual bacterial abundances within each sample. Genera boxed in red represent those found within DMC-18. Genera found within DMC-18, as well as detected in stool from mice exposed to DMC-18 were used to formulate the refined community, DMC-4.

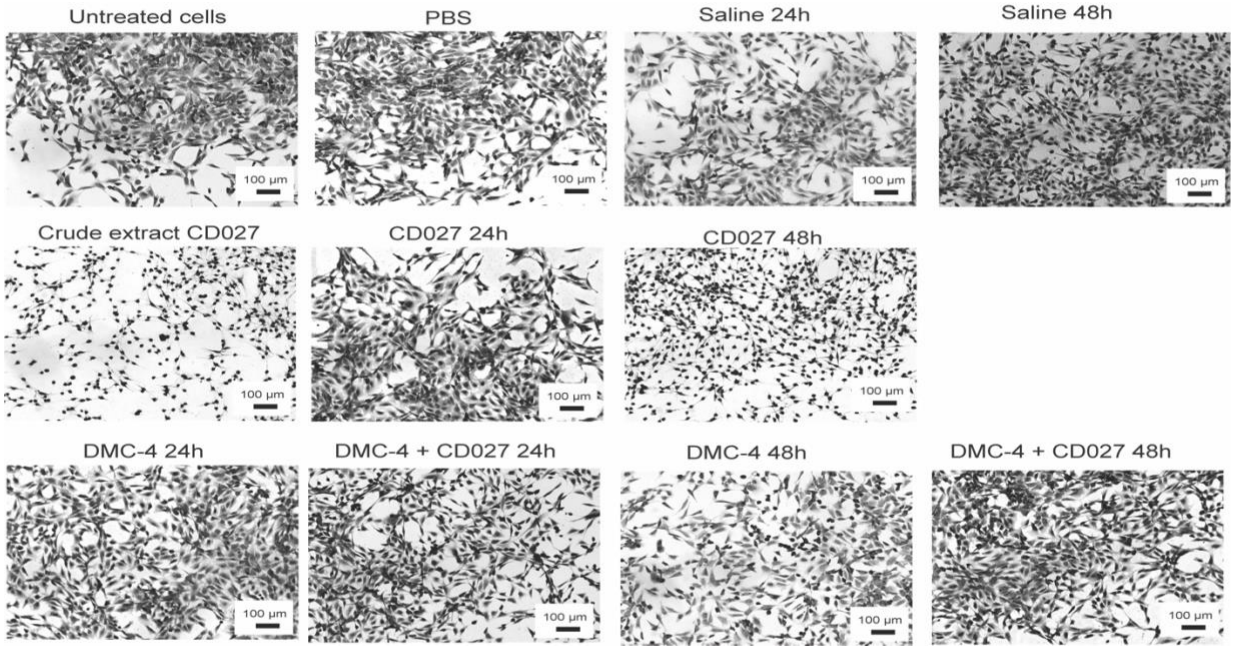

Supplementary Figure 3: Representative images of fibroblasts cultures stained with Giemsa that were incubated with stool extract from mice pre-exposed to DMC-4 and infected with CD027. All experiments were performed including CD027 crude extracts and stool extracts from CD027- infected mice as positive controls; PBS, DMC-4 stool extracts as negative controls and untreated cells as reference.

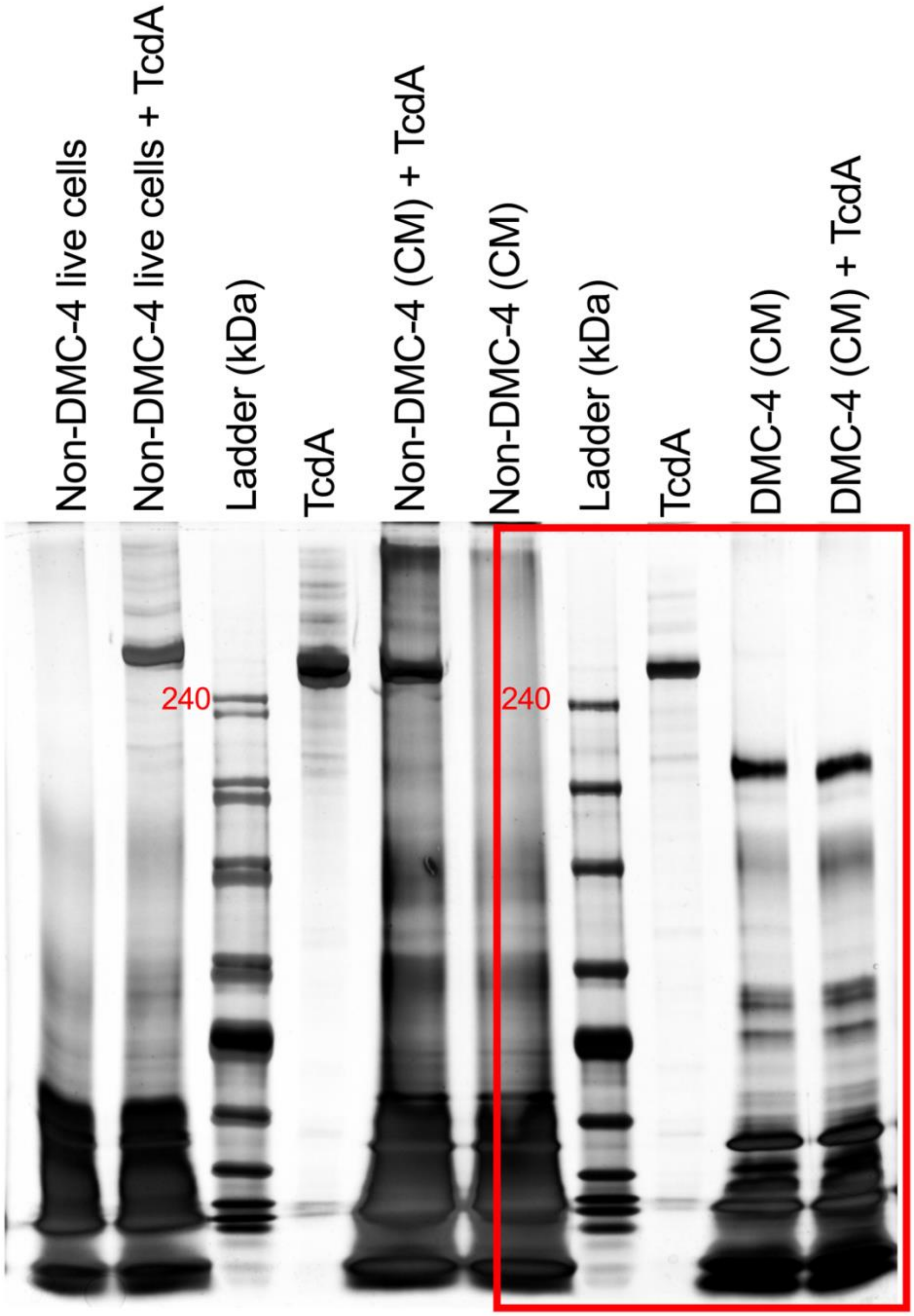

Supplementary Figure 4. Uncropped gel image for Fig.5 **b**.

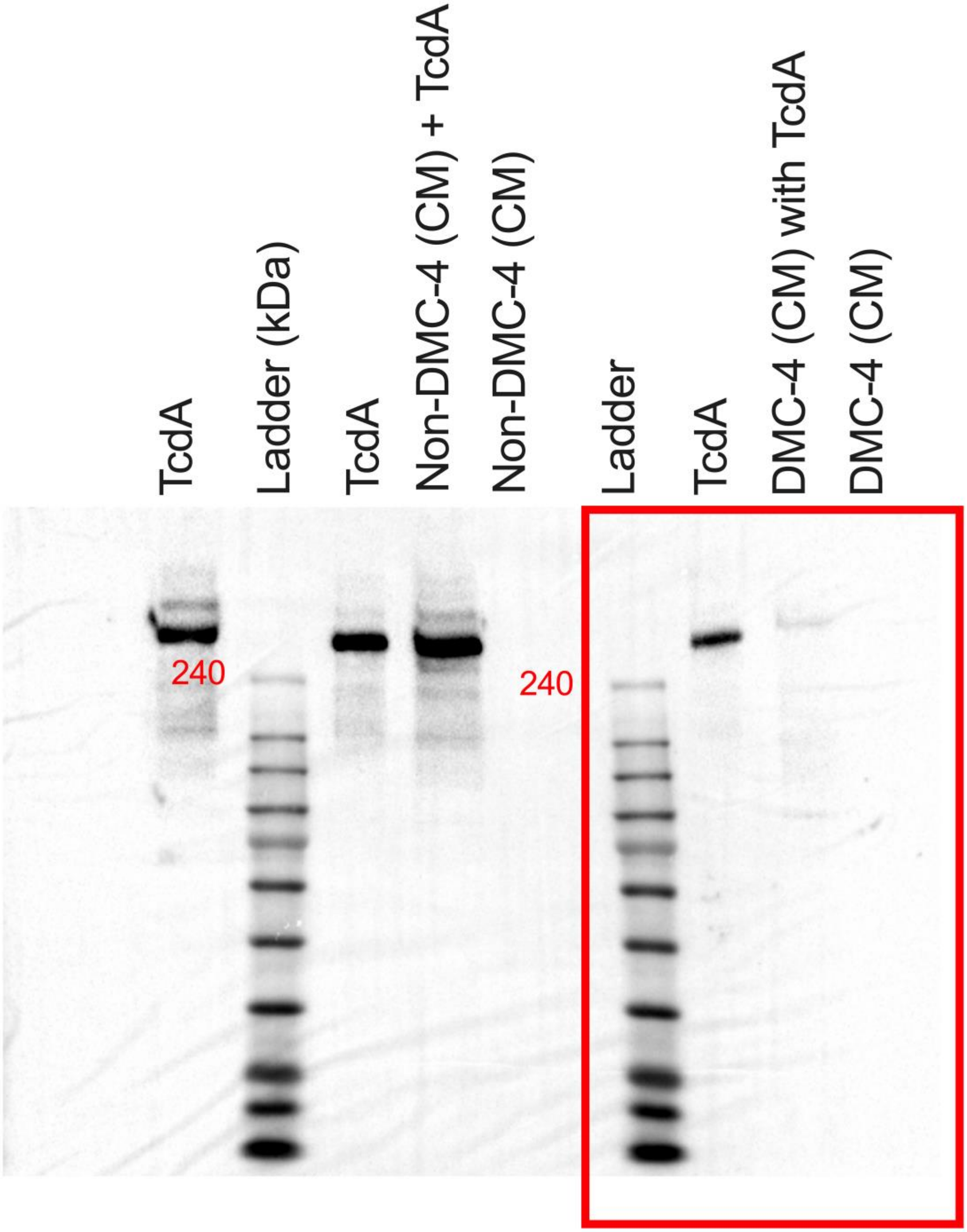

Supplementary Figure 5. Uncropped gel image for Fig.5 **c**.

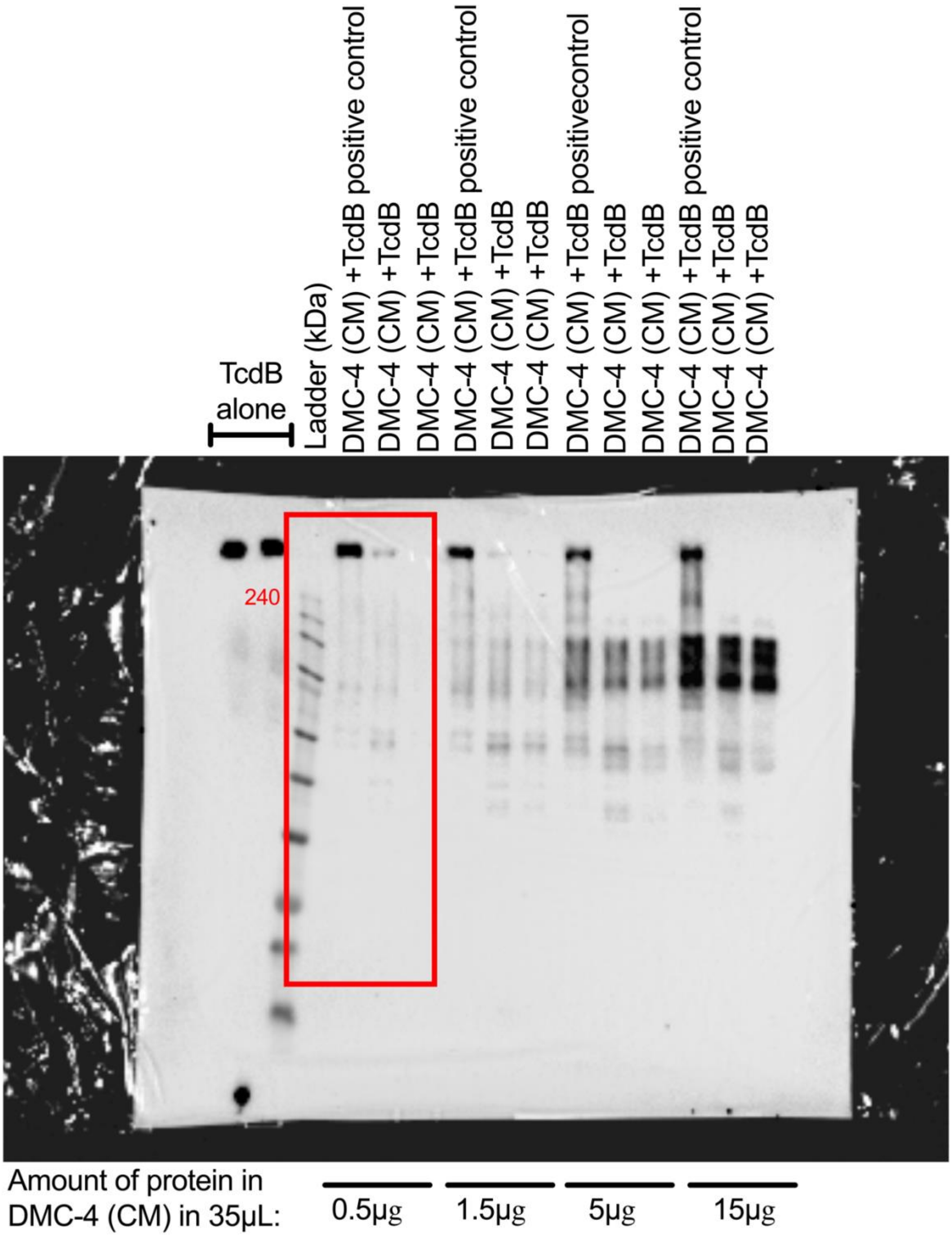

Supplementary Figure 6. Uncropped gel image for Fig.5 **d**.

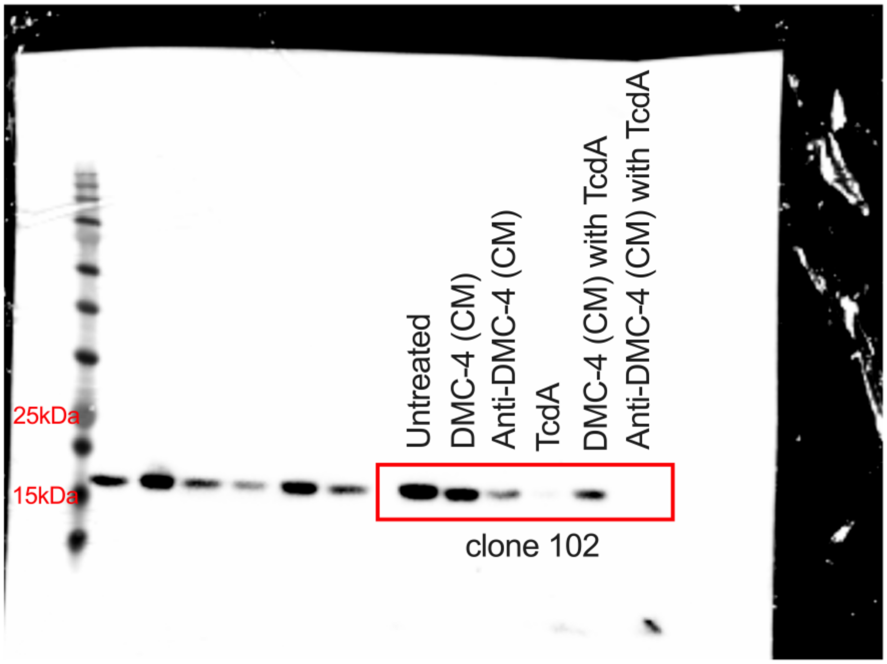

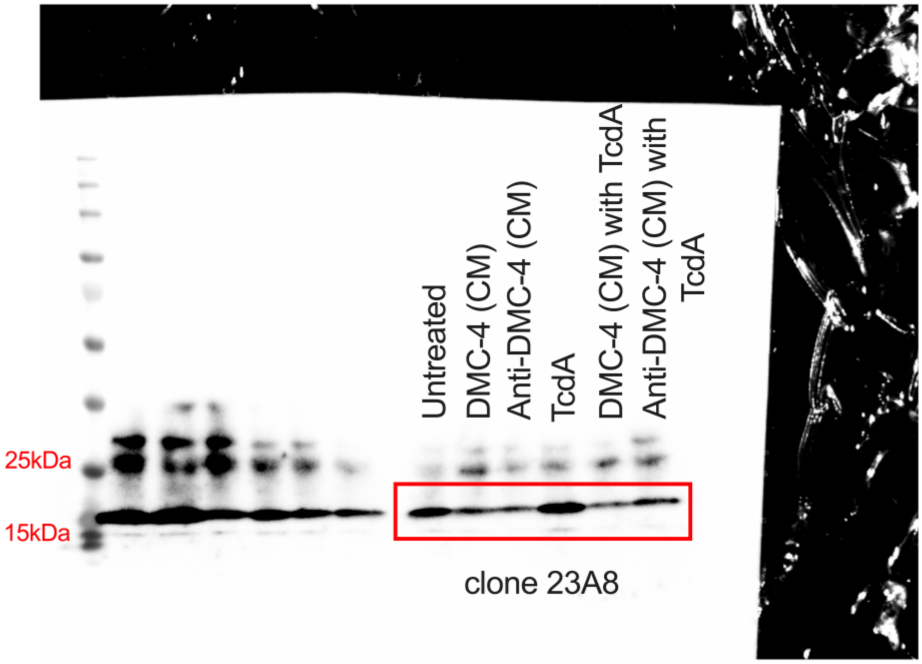

Supplementary Figure 7. Uncropped gel images for Fig.5 **e**.
