# Supplementary material for: Defined microbial communities and their soluble products protect mice from Clostridioides difficile infection
Source: Commun Biol. 2024 Jan 27;7:135. doi: 10.1038/s42003-024-05778-6 (PMC10821944; doi:10.1038/s42003-024-05778-6)
Supplement: Supplementary file 4 — Reporting Summary [file 42003_2024_5778_MOESM4_ESM.pdf]

nature portfolio

Corresponding author(s): Prameet Sheth (ps70@queensu.ca)

Last updated by author(s): Dec 12, 2023

Reporting Summary

Nature Portfolio wishes to improve the reproducibility of the work that we publish. This form provides structure for consistency and transparency in reporting. For further information on Nature Portfolio policies, see our Editorial Policies and the Editorial Policy Checklist.

Statistics

For all statistical analyses, confirm that the following items are present in the figure legend, table legend, main text, or Methods section.

n/a	Confirmed	
☐	☒	The exact sample size (*n*) for each experimental group/condition, given as a discrete number and unit of measurement
☐	☒	A statement on whether measurements were taken from distinct samples or whether the same sample was measured repeatedly
☐	☒	The statistical test(s) used AND whether they are one- or two-sided<br>*Only common tests should be described solely by name; describe more complex techniques in the Methods section.*
☒	☐	A description of all covariates tested
☐	☒	A description of any assumptions or corrections, such as tests of normality and adjustment for multiple comparisons
☐	☒	A full description of the statistical parameters including central tendency (e.g. means) or other basic estimates (e.g. regression coefficient) AND variation (e.g. standard deviation) or associated estimates of uncertainty (e.g. confidence intervals)
☐	☒	For null hypothesis testing, the test statistic (e.g. *F*, *t*, *r*) with confidence intervals, effect sizes, degrees of freedom and *P* value noted<br>*Give P values as exact values whenever suitable.*
☒	☐	For Bayesian analysis, information on the choice of priors and Markov chain Monte Carlo settings
☒	☐	For hierarchical and complex designs, identification of the appropriate level for tests and full reporting of outcomes
☒	☐	Estimates of effect sizes (e.g. Cohen's *d*, Pearson's *r*), indicating how they were calculated

Our web collection on statistics for biologists contains articles on many of the points above.

Software and code

Policy information about availability of computer code

Data collection	No software was used.
Data analysis	16s rRNA Sequencing analysis: Raw sequencing read quality was assessed using the tool FastQC v0.11.9. Low quality nucleotides and adapter sequences were removed using Cutadapt v3.4 within the TrimGalore v0.6.6 wrapper. 16S rRNA analysis and classification were performed using the Quantitative Insights into Microbial Ecology (QIIME 2 2019.7.0) software package. Reads were processed with DADA253 for quality filtering (forward and reverse reads were trimmed at 270 and 245 bases, respectively), denoising, and chimera removal. All sequencing reads were inserted into a reference phylogenetic tree using SATé-enabled phylogenetic placement and taxonomy was assigned to each read using the classify-sklearn machine learning classifier against the Greengenes 13.8 99% operational taxonomic units reference sequences. The taxonomy table was imported into R version 4.2.1 (R Project for Statistical Computing) for visualization using ggplot2 version 3.4.0 and RColorBrewer version1.1-3.<br><br>All other data analysis: Data were analyzed using GraphPad Prism Version 9.5 for Macintosh, GraphPad Software (USA). The results are expressed as the mean ± of the standard error of the mean (SEM). Eight mice were used per group (n=8). Data were analyzed using a t-Test with Mann-Whitney, two-tailed test. Ordinary one-way analysis of variance (ANOVA) with Dunnett's or Tukey's test was performed to compare the difference between the means of more than two groups. A two-way ANOVA with Tukey's test was performed to analyze the interrelationship of two independent variables. T-test with Kruskal-Wallis and Dunn's test. p<0.05 was chosen to reject the null hypothesis.

For manuscripts utilizing custom algorithms or software that are central to the research but not yet described in published literature, software must be made available to editors and reviewers. We strongly encourage code deposition in a community repository (e.g. GitHub). See the Nature Portfolio guidelines for submitting code & software for further information.

Data

Policy information about availability of data

All manuscripts must include a data availability statement. This statement should provide the following information, where applicable:

- Accession codes, unique identifiers, or web links for publicly available datasets
- A description of any restrictions on data availability
- For clinical datasets or third party data, please ensure that the statement adheres to our policy

All data associated with this study are available in the main text or Supplementary Materials.

Research involving human participants, their data, or biological material

Policy information about studies with human participants or human data. See also policy information about sex, gender (identity/presentation), and sexual orientation and race, ethnicity and racism.

Reporting on sex and gender	N/A
Reporting on race, ethnicity, or other socially relevant groupings	N/A
Population characteristics	N/A
Recruitment	N/A
Ethics oversight	N/A

Note that full information on the approval of the study protocol must also be provided in the manuscript.

Field-specific reporting

Please select the one below that is the best fit for your research. If you are not sure, read the appropriate sections before making your selection.

☒ Life sciences ☐ Behavioural & social sciences ☐ Ecological, evolutionary & environmental sciences

For a reference copy of the document with all sections, see nature.com/documents/nr-reporting-summary-flat.pdf

Life sciences study design

All studies must disclose on these points even when the disclosure is negative.

Sample size	The sample size for this study was determined based on published literature in the field and preliminary experimentation. No sample calculation was performed.
Data exclusions	No data was excluded from this study.
Replication	In vivo experiments were done with replicates in each independent run Experiments were done consisting of a minimum of n = 3 mice in each run and were replicated. Results are presented with each dot in the plots representing an individual mouse. All 'n' numbers are provided in the figure legends of each figure when appropriate. In vitro assays were replicated n=3 times.
Randomization	Mice were randomly selected to be in each of the groups. This was not based on any criteria.
Blinding	No blinding was done as each group had a different intervention performed.

Reporting for specific materials, systems and methods

We require information from authors about some types of materials, experimental systems and methods used in many studies. Here, indicate whether each material, system or method listed is relevant to your study. If you are not sure if a list item applies to your research, read the appropriate section before selecting a response.

Materials & experimental systems

n/a	Involved in the study
☐	☒ Antibodies
☐	☒ Eukaryotic cell lines
☒	☐ Palaeontology and archaeology
☐	☒ Animals and other organisms
☒	☐ Clinical data
☒	☐ Dual use research of concern
☒	☐ Plants

Methods

n/a	Involved in the study
☒	☐ ChIP-seq
☒	☐ Flow cytometry
☒	☐ MRI-based neuroimaging

Antibodies

Antibodies used

Antibodies used in this paper:
anti-Rac1 (102):mouse monoclonal, BD Transduction Laboratories (cat: 610650, lot:3200771)
anti-Rac1(23A8): and Millipore Sigma (cat:05-389, lot:2197496)
anti-TcdA: mouse monoclonal antibody, Santa Cruz Biotechnology (cat: PCG4, lot: 12909)
anti-TcdB: sheep polyclonal antibody R&D Systems (cat:AF6246, lot: CETN0119101)
goat anti-mouse HRP: Invitrogen (cat:31430, lot: X13341610)
donkey-anti-Sheep HRP: Sigma-Aldrich, USA (cat:A3415, lot: SLBB9255)

Validation

All antibodies were used according to manufacturer's instruction.

Eukaryotic cell lines

Policy information about cell lines and Sex and Gender in Research

Cell line source(s)

NIH 3T3 fibroblasts (ATCC)

Authentication

N/A

Mycoplasma contamination

Cell lines were not tested for Mycoplasma contamination. ATCC certifies that there is no Mycoplasma contamination detected in this cell line.

Commonly misidentified lines
(See ICLAC register)

No commonly misidentified lines were used in this study.

Animals and other research organisms

Policy information about studies involving animals; ARRIVE guidelines recommended for reporting animal research, and Sex and Gender in Research

Laboratory animals

Six-week-old C57BL/6 female mice from Jackson Laboratory (USA) were used in this study.

Wild animals

This study did not involved wild animals.

Reporting on sex

Only female mice were used in this study as per the requirement for males to be individually housed due to inter-species aggression. Since females can be housed up to 5 per cage, it allows us to increase our biological replicates while ensuring there is no cage-to-cage variability.

Field-collected samples

This study did not involve samples collected from the field.

Ethics oversight

This study was carried out in accordance with the Canadian Council of Animal Care guidelines and approved by the Queen's University Animal Care Committee and the Biosafety committee.

Note that full information on the approval of the study protocol must also be provided in the manuscript.
